# Association between Nutritional Status and Treatment Response and Survival in Patients Treated with Immunotherapy for Lung Cancer: A Retrospective French Study

**DOI:** 10.3390/cancers14143439

**Published:** 2022-07-15

**Authors:** Manon Gouez, Lidia Delrieu, Carole Bouleuc, Nicolas Girard, Bruno Raynard, Timothée Marchal

**Affiliations:** 1 Department of Prevention Cancer Environment, Léon Bérard Cancer Centre, 69008 Lyon, France; manon.gouez@lyon.unicancer.fr; 2 Residual Tumour & Response to Treatment Laboratory, RT2Lab, Translational Research Department, INSERM, U932 Immunity and Cancer, Institut Curie, Paris University, 75005 Paris, France; lidia.delrieu@curie.fr; 3 Department of Supportive Care, Institut Curie, 75005 Paris, France; carole.bouleuc@curie.fr; 4 Institut Curie, Institut du Thorax Curie Montsouris, 75005 Paris, France; nicolas.girard@curie.fr; 5 Department of Supportive Care, Unité Transversale de Diététique et de Nutrition Centre Gustave-Roussy, 94800 Villejuif, France; bruno.raynard@gustaveroussy.fr

**Keywords:** advanced cancer, immunotherapy, lung cancer, nutritional status, survival

## Abstract

**Simple Summary:**

It is estimated that 73% of advanced non-small cell lung cancers (NSCLC) will become malnourished and develop cachexia which is considered as an independent prognostic factor. Therefore, this study aimed to investigate the association between nutritional assessments and (i) immunotherapy efficacy, (ii) tolerance, and (iii) survival in patients with an advanced NSCLC stage of lung cancer treated with immunotherapy. In total, 67% of the 120 patients analysed were not malnourished, 20% presented with moderate malnutrition, and 13% presented with severe malnutrition. There was no significant link between the nutritional status and the toxicity of immunotherapy. However, severe malnutrition was significantly associated with treatment efficacy and with a lower survival rate. Malnutrition appears to have a negative impact in the case of immunotherapy, in contrast to a high body mass index, which seems to be protective. In addition to confirming the benefits of early and appropriate nutritional management, research must also focus on catabolism and the uncontrolled inflammatory mechanisms.

**Abstract:**

Malnutrition is associated with a greater risk of morbidity and mortality and lower tolerance to chemotherapy. Our purpose was to study the association between nutritional status and the efficiency and tolerance of immunotherapy in non-small cell lung cancer (NSCLC). Nutritional and oncological data were reported at 2 months (M2) and 4 months (M4) after the initiation of immunotherapy (M0). The influence of nutritional status at M0 was estimated with the efficacy and toxicity of immunotherapy at M2 to M4. In total, 127 patients were included in the study, and nutritional status was estimated at M0 for 120 patients: 67% were not malnourished, 20% presented with moderate malnutrition, and 13% presented with severe malnutrition. There was no significant link between the nutritional status at M0 and the toxicity of immunotherapy at M2 and M4. However, severe malnutrition was significantly associated with treatment efficacy at M2 (*p* = 0.04) and with a lower survival rate with an HR (Hazard Ratio) = 2.32–95% C.I: 1.13–4.75 (*p* = 0.02). Furthermore, a monthly decrease of 1% of the weight had an HR = 1.17–95% C.I: 1.13–1.21 (*p* = 0.0001). Severe malnutrition and weight loss are independent factors associated with lower survival. Studies integrating the systemic detection of sarcopenia with a closer nutritional follow-up could highlight an improvement in survival.

## 1. Introduction

Lung cancer is a leading cause of cancer deaths worldwide, with an estimated 1,796,144 deaths in 2020 [1]. More than half of non-small cell lung cancers (NSCLCs) are diagnosed at an advanced stage [2]. Moreover, patients with a metastatic NSCLC have a high symptom burden and poor quality of life. Estimated prognosis is less than 1 year [3,4].

The advent of immune checkpoint inhibition with anti-PD-1 and anti-PD-L1 has fundamentally changed the management of patients with advanced NSCLC [5]. The choice of treatment for lung cancer depends on the histology, stage, and mutation status. Immunotherapy is indicated as a second-line treatment in patients with advanced squamous and non-squamous NSCLC, without activating mutations, previously treated with platinum-based doublet chemotherapy or alternative first-line agents [6,7]. As an example, immunotherapy significantly prolonged overall survival (OS), with a 2-year OS rate of 48% —regardless of PD-L1 expression—and a favourable safety profile compared with docetaxel, which was the only reference treatment for these patients [2].

Despite these therapeutic advances, metastatic lung cancer has a negative impact on patients’ physical, psychological, and social functioning. Patients reported symptoms due to disease and treatment, including fatigue, decreased appetite, nausea, and diarrhoea [2]. Nutrition is an important therapeutic component. Studies suggest a link between malnutrition and the tolerance and/or efficacy of chemotherapy [8]. The prevalence of malnutrition is high in patients with cancer, especially at an advanced stage, and it is estimated that 73% of advanced NSCLC will become malnourished and develop cachexia [9,10]. Weight loss has been identified as an independent prognostic factor, regardless of the cancer site and disease severity [11]. Moreover, cachexia is responsible for over 20% of all cancer-related deaths [12]. Malnutrition is a prognostic factor of poor treatment outcomes, correlated with a decreased quality of life and an increased chemotherapy toxicity [13]. Beyond that, nutritional care can improve outcomes, but is under-evaluated in daily practices [14], and little is known about the link between malnutrition and immunotherapy. Therefore, this study aimed to investigate the association between nutritional assessments and (i) immunotherapy efficacy, (ii) tolerance, and (iii) survival in patients with an advanced NSCLC stage of lung cancer treated with immunotherapy.

## 2. Materials and Methods

### 2.1. Study Design and Setting

We conducted a retrospective analysis in a cohort selected from one comprehensive cancer centre. In this centre, nutritional care is triggered by oncologists when they detect nutritional risk factors or malnutrition. Nutritional care is available for in- and outpatients, delivered by specialised healthcare professionals composed of five dieticians and one nutritionist physician.

### 2.2. Study Population and Data Source

The cohort was selected using ConSoRe, a new data analysis solution aggregating diverse forms of structured and unstructured data extracted from digital medical files at several French cancer centres. ConSoRe uses natural language processing to search aggregated data and perform advanced data mining [15]. This data mining tool was used to find all patients treated at Institut Curie for all stages of lung cancer.

To be included, patients had to be treated at the Institut Curie for lung cancer of any stage. They should have received at least two injections of second-line immunotherapy with anti-PD-1/PD-L1 inhibitors between June 2015 and November 2017 in a clinical trial or not (mostly nivolumab) at the time the study was conducted. The inclusion date was the date of the first nivolumab injection, and follow-up ended either at the date of death or in May 2018. We excluded patients who had had other previous cancers.

### 2.3. Data Collection

Data collection was approved on 10 April 2018 by the local data protection officer on behalf of French regulatory authorities (Commission Nationale de l’Informatique et des Libertés, CNIL) in accordance with MR004 methodology. All patients were informed of the possibility of their health data being used for research purposes and expressed no opposition to this possibility. Parameters were assessed at baseline (i.e., first dose of immunotherapy) (M0), two months after the first dose of immunotherapy (M2), and at four months (M4).

#### 2.3.1. Patient Characteristics

Clinical data and tumour characteristics were extracted from digital medical files, including hospitalisation and consultation notes. At baseline, sex, date of birth, height, body weight (kg), calculated body mass index (BMI; kg/m^2^), and performance status (PS) were collected. Concerning the disease, the personal history of the lung cancer integrated site and number of metastases, current treatment, date of first immunotherapy administration, presence of comorbidities (cardiac, respiratory, and metabolic), and PD-L1 status (considered positive if >1%) were collected.

#### 2.3.2. Tumour Outcomes

The Response Evaluation Criteria in Solid Tumours (RECIST) V1.1 was used to assess tumour progression between diagnosis and the second and fourth months. Therapeutic choices included the pursuit of immunotherapy, change in treatment line, or palliative care.

#### 2.3.3. Toxicity

Treatment toxicities were noted according to the National Cancer Institute’s Common Terminology Criteria for Adverse Events (NCI-CTCAE) v5.0. Grade and type of toxicity were identified by examining all hospitalisation reports and oncology visit reports recorded in the patient’s medical file.

#### 2.3.4. Malnutrition: Clinical Data

The nutritional status of each patient was defined according to French Health Authority guidelines. The values retained in this study were those commonly used to diagnose malnutrition: a weight loss of 5% in one month or 10% in six months defined moderate malnutrition (along with BMI < 18.5), and 10% in one month or 15% in six months defined severe malnutrition (along with BMI ≤ 17). Date of nutritional follow-up, dietary intake (only total calories ingested), causes of dietary intake decrease (appetite disorders, digestives obstruction, digestive disorder, restrictive diet, multi-medication, or other reasons), and usual weight were collected.

Biological parameters (pre-albumin, albumin, C-reactive protein, and immune markers) were assessed by clinical dietitians affiliated with the study.

The modified Glasgow Prognostic Score (mGPS) was calculated according to the method of Forrest et al. [16]. Patients with a normal albumin level (3.5 g/dL) and CRP (1.0 mg/dL) were allocated a score of 0, and those with both low albumin (<3.5 g/dL) and high CRP (>1.0 mg/dL) were scored 2 [17]. Patients with only an elevated CRP (>1.0 mg/dL) were assigned a score of 1.

### 2.4. Statistical Analyses

Associations between clinical characteristics and nutritional status were studied with chi square or Fisher’s exact tests for categorical variables and Student’s *t*-tests for continuous variables. Predictive factors of treatment response or toxicity were identified by univariable analysis using chi square or Fisher’s exact tests for categorical variables and logistic regression with multivariable fractional polynomials (MFPs) for continuous variables. Multivariable analysis was performed using logistic regression. Concerning the analysis of the response rate at M2, the parameters were estimated with a penalised likelihood due to the complete separation of data.

For survival analysis, the prognostic value of categorical predictors was studied with log-rank tests. The effect of the weight variation over time on survival was studied by means of a joint model. Weight variation was modelled with a linear mixed model, while survival was modelled with a Cox model. Dietician consultations were treated as time-dependent covariates. Continuous variables were studied by means of the MFP. Multivariable analysis used a joint model.

Statistical analyses were conducted with R software. Values of *p* < 0.05 were considered to indicate significance in all statistical tests.

## 3. Results

### 3.1. Patient Characteristics

#### 3.1.1. General Characteristics

**Figure 1:**
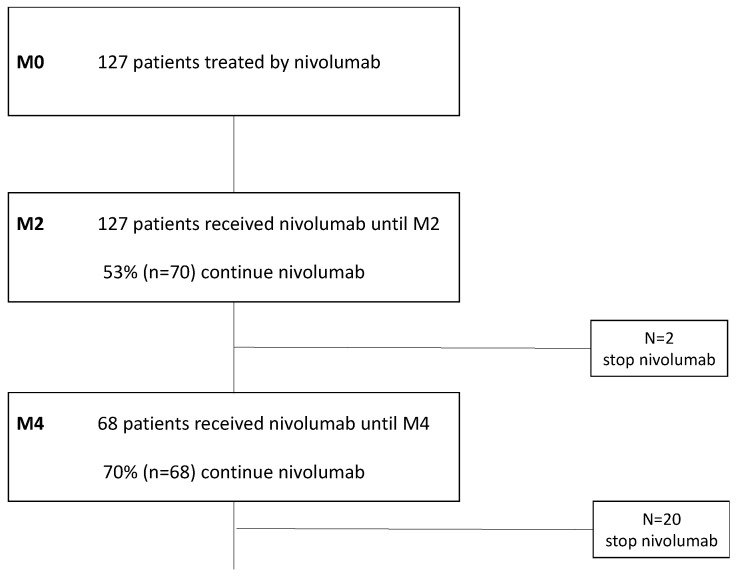
Flow chart describing the selection of the study population using ConSore (n = 127).

Patient characteristics are shown in Table 1. In total, 127 patients treated with immunotherapy at Institut Curie (Paris, France) were included between 18 June 2015 and 16 November 2017 (Figure 1). The mean age at first injection was 67 years (Sd = 8), and 56% (n = 71) of patients were men. The mean time since cancer appeared was 16 months. Adenocarcinoma was the most frequent histological type, found in 71% of cases. Almost all the patients included had stage IV disease (92.9%; n = 116), metastatic in at least one site in 93% of cases (n = 118) and two sites in 68% (n = 86). For 84 patients, immunotherapy was administered as second-line treatment, and 98% of these had a platinum-based chemotherapy as first-line treatment.

#### 3.1.2. Nutritional Status at Baseline

A total of 13% (n = 16) of patients suffered from severe malnutrition, and 20% (n = 24) suffered from moderate malnutrition. The mean BMI at the start of immunotherapy was 23.14 kg/m^2^, and approximately 27% (n = 32) of patients had weight loss greater than 10% compared to the usual weight.

#### 3.1.3. Dietary Management

When immunotherapy was introduced, approximately half of the patients were followed by a dietician: 40% had a decrease in food ingesta, and the most common reason was loss of appetite.

### 3.2. Association between Nutritional Status and Clinical Characteristics

The majority (90%) of patients had a performance status (PS) of 0–1 at the initiation of immunotherapy. We found an association between PS and smoking status at inclusion and nutritional status (*p* = 0.003 and *p* = 0.007, respectively).

### 3.3. Influence of Nutritional Status on Immunotherapy Efficacy 

All included patients received immunotherapy until M2 (n = 127). A total of 60.6% of patients progressed during the 2 months (n = 77), and 39.3% had a stable or regressing disease (n = 50). Immunotherapy was continued for a total of 55.1% of included patients (70 of 127 patients) at the end of M2. Grade I or II toxicities occurred in 15% (n = 19) of patients, with only 1 experiencing grade III toxicity. Of the 127 patients included, 53% (n = 68) received immunotherapy until at least M4, and the majority (70%) continued to be treated beyond M4. 

In patients who had regression of their disease (22 out of 127 individuals included, i.e., about 17%), severe undernutrition was significantly associated with therapeutic efficacy at M2 (*p* = 0.04) in univariate analysis, as well as at M4 (*p* = 0.05). In multivariate analysis, no factor was significantly associated with therapeutic efficacy.

### 3.4. Influence of Nutritional Status on Immunotherapy Tolerance

**Figure 2:**
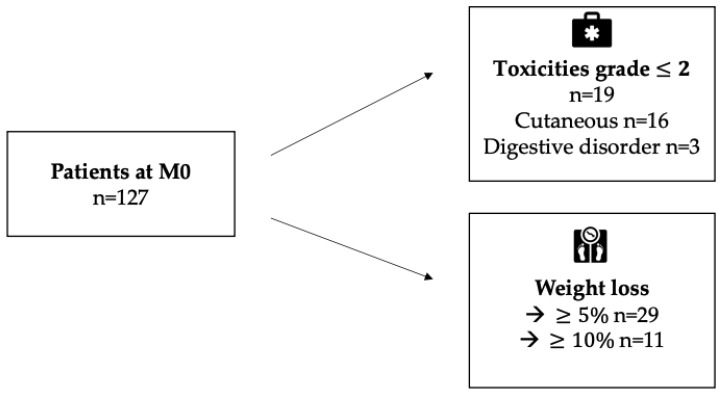
Onset of toxicities and evolution of weight loss at M2 (n = 127).

Only 15% of the 127 patients experienced toxicities at M2, almost exclusively grade II or less (94.7%) (Figure 2). The most common toxicity was cutaneous. Only 3 had digestive toxicities. 

In multivariate analysis, smoking status, the occurrence of metastasis, and nutritional status were not associated with tolerance or efficacy of immunotherapy.

### 3.5. Influence of Nutritional Status on Survival

Median OS was 13 months (95% CI: 10–18) for the whole population, and the median follow-up was 17 months.

**Figure 3:**
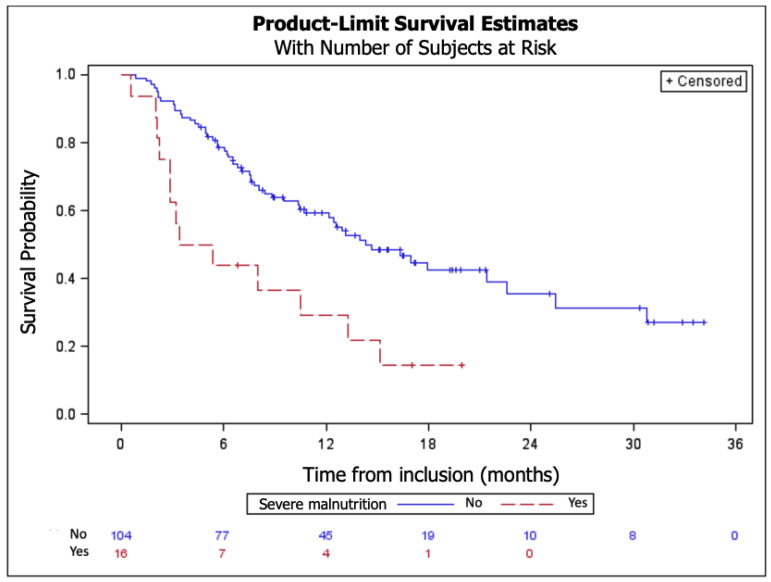
Influence of nutritional status (severe malnutrition yes/no) on survival (*p* = 0.02) (n = 127).

At univariate analysis, severe malnutrition and weight loss were associated with reduced survival (HR = 2.32, IC 1.13–1.21, *p* = 0.02) (Figure 3).

A multivariate analysis was then performed with the variables of the univariate model including performance status, weight change, and severe malnutrition at the beginning of treatment. Severe malnutrition at baseline (M0) (HR = 2.32, 95% CI 1.13–4.75, *p* = 0.02), performance status (HR = 1.82, 95% CI 0.95–3.49), and weight change corresponding to a 1% monthly weight decrease compared to weight at baseline (HR = 1.16 95% CI 1.12–1.21, *p* < 0.01) were associated with lower survival.

### 3.6. Nutritional Assessment, Weight Changes during Treatment

**Weight evolution.** Of the 127 patients at inclusion, 124 had a weight measurement at M2. Of them, 23% (29 patients) experienced more than 5% weight loss compared to their weight at M0, including 11 patients with a weight loss of more than 10%.

**Dietary management.** There was no relation between dietary management and reduction in food ingesta.

Furthermore, the results showed an association between decreased ingesta and dietary management during follow-up in patients not initially managed (at M0) (HR = 3.8, IC 1.8–6.3, *p* < 0.001).

## 4. Discussion

The main objective of this retrospective study was to evaluate the association between nutritional assessments and (i) immunotherapy efficacy, (ii) tolerance, and (iii) survival in patients with an advanced stage of lung cancer treated with immunotherapy. Although severe malnutrition was significantly associated with treatment efficacy at M2, there was no significant relationship between nutritional status and toxicity. However, a weight loss of more than 1% compared with baseline was associated with a poorer OS.

In current practice, several biomarkers are used to predict the response to immune checkpoint inhibitors, such as PD-ligand 1 (PD-L1) expression level and tumour genetics [18,19]. However, these biomarkers have poor reproducibility, and immunotherapy is an expensive therapy that may cause adverse effects for our patients [20,21]. New predictive biomarkers are necessary to optimise the therapeutic benefits for patients and minimise the risk of toxicity [22].

Weight loss is a common symptom that is one of the first symptoms of cancer in more than 50% of cases and occurs in more than 70% of cases during the course of the disease [23]. Weight loss is multifactorial, but anorexia and hypermetabolism caused by tumour activity are the two main causes of weight loss. Pain (abdominal or not), sleep, mood and transit disorders, nausea, and vomiting are all symptoms that stimulate the anorexigenic pathway at the expense of the orexigenic pathway. The same applies to taste and smell disorders, which are sometimes present at diagnosis in 15% to 20% of patients and whose prevalence remains high, up to 39% for dysgeusia [24,25]. In parallel, tumour metabolism and inflammation can increase resting energy expenditure and simultaneously decrease energy intake, tilting the weight balance toward a negative energy balance, and thus toward weight loss [26]. TGF-β super family cytokines (GDF11, activins) can lead to a decrease in protein synthesis through a cellular cascade and a significant increase in muscle protein breakdown [27,28]. Finally, the pro-inflammatory cytokines TNF-a and IL-1 directly activate certain pathways, leading to muscle proteolysis. Moreover, TNF-a is also a potent stimulant of the anorexigenic pathway, and IL-1 acts negatively on the orexigenic pathway, reducing protein-energy intake limiting muscle protein synthesis [29,30].

Although complex, there is a relationship between the immune system and the nutritional status of patients, which may influence the response to immune checkpoint inhibitors and thus affect tumour progression and prognosis [31,32]. Malnutrition in cancer is a major problem whose diagnosis relies on the combination of a phenotypic and an aetiological criterion. Furthermore, cancer cachexia is characterised by an inflammatory state causing immune and nutritional disorders [32]. This condition disrupts T-cell metabolism and function through several interrelated cytokines, such as IL-6 and TNF-α, as well as stress hormones, and has been shown to be associated with a poor response to immunotherapy [33]. On the other hand, prolonged exposure to immunotherapy appears to be a factor in better response to treatment [34]. One study showed that cancer patients with more advanced cachexia had lower serum concentrations of immunotherapy [35]. Recently, other studies have found that the progression of cachexia decreases the efficacy of immunotherapy treatment in patients with NSCLC and gastric cancer [36,37]. The present study corroborates these data, as nearly all patients included in this study had advanced and long-standing disease, and severe malnutrition was significantly associated with a decrease in the efficacy of immunotherapy at M2 and M4 in the univariate analysis. This effect disappeared in multivariate analysis, probably due to a lack of power. However, it remains important to consider the impact of metabolic abnormalities caused by malnutrition reducing the therapeutic effect of immune checkpoint inhibitors [38,39]. 

In our study, no direct link between nutritional status and tolerance to immunotherapy could be demonstrated. This may be explained by the specific functioning of immunotherapy, which aims to restore the immune system’s detection capacity with respect to tumour cells, in order to activate the apoptotic cascade and thus stop tumour proliferation.

Several studies confirm our observations regarding the statistically significant relationship between survival and severe malnutrition on the one hand, and between survival and weight loss on the other [8,38]. While Degens et al. [40] found that an early weight loss of more than 2% was an independent predictor of poor overall survival, our study shows that even a minimal loss of 1% per month is associated with poorer survival. These elements strongly suggest that sarcopenia should be detected earlier, especially as patients are seen regularly in consultation and benefit from complementary examinations that can be used for diagnosis (biological and scannographic examinations, in particular). Early management, in parallel to the cancerous disease, must be carried out as soon as the diagnosis of sarcopenia is made, and regular follow-up is then necessary. In our study, the prevalence of malnutrition at initiation of immunotherapy was 33%. This figure is slightly lower than in the literature and is probably underestimated due to a lack of data, or to an overestimation of the weight declared by the patients [41]. In contrast, almost half of the patients had received at least one consultation with a dietician. Unfortunately, we do not have a precise description of the dietary management in our study, which could impact differently on the management of patients. Thus, it is possible that the nutritional measures implemented during the consultations prior to the introduction of immunotherapy may have helped curtail any weight loss, thereby correcting the nutritional status. Simply correcting weight loss, i.e., maintaining a stable weight by specific nutritional measures (e.g., meal enrichment, use of oral nutritional supplements), could increase survival without disrupting the specific course of treatment [42,43]. The objective is to increase the patient′s caloric and protein intake through everyday foods, considering all other aspects of the diet [44]. The effectiveness of oral nutritional supplements has been demonstrated in various situations of malnutrition or at risk, and their prescription is recommended in particular for the elderly and for cancer patients [45,46]. Several randomised controlled trials, systematic reviews, and meta-analyses have shown that nutritional care improves weight status, energy and protein intakes, treatment tolerance, and survival, and reduces nutrition-related symptoms, hospital readmissions, and mortality in patients with different tumour types undergoing various cancer treatments [47,48,49,50,51]. Just as it seems to be accepted for pain, it would also be desirable to have a common discourse between carers reinforcing the action on nutritional management to improve its quality and ensure the patient′s adherence to this therapeutic aspect [52].

Nutritional assessment, however, should not be limited to biological or anthropometric data, as is the case in our study. The analysis of body composition on lumbar CT scans becomes important as it allows the quantification of skeletal muscle mass (*SMM*) and total, visceral, and subcutaneous adipose tissue. Indeed, early loss of muscle mass during immunotherapy treatment, independent of weight changes, does not appear to be predictive of overall survival in contrast to patients treated with chemotherapy [40]. Conversely, several studies, including the one conducted by Cortellini et al., have shown that there is a significant association between obesity (BMI > 25) and improved ICI scores in 976 patients [53]. Similarly, recent evidence suggests that the presence of adipose (white) tissue may influence the response to immune checkpoint inhibitors in patients with advanced cancer. Although these techniques are very useful, the topography to be analysed (L1, L3, psoas, or skeletal muscles?) and the cut-offs used need to be refined.

## 5. Conclusions

In conclusion, cachexia remains an important factor associated with survival in cancer patients, regardless of the treatment received. Pre-treatment weight loss and low body mass index are well known to adversely affect prognostic features in cancer patients treated with chemotherapy [11,54]. Malnutrition also appears to have a negative impact in the case of immunotherapy, in contrast to a high BMI, which seems to be protective. New biomarkers are currently being studied, such as the Prognostic Nutrition Index (PNI), calculated from serum albumin and total lymphocyte count. A meta-analysis has shown that the PNI is a useful indicator for assessing nutritional and immunological conditions in lung cancer [55]. PNI is an inexpensive, easy-to-perform, non-invasive, rapid, and standardised tool for estimating cancer prognosis [56].

Prognostic estimation is therefore crucial in cancer management, as is early assessment of nutritional status, as it can have a significant influence on the choice of therapeutic approach. In addition to confirming the benefits of early and appropriate nutritional management, research must also focus on catabolism and the uncontrolled inflammatory mechanisms. This approach would offer a second aspect of action, potentiating the effect of increased nutritional intake, better used by the body.

## Figures and Tables

**Table 1:** Sociodemographic and clinical characteristics of the study population at baseline (n = 127).

Characteristic	Mean (SD)	*n* (%)
Sex Men Women		71 (56) 56 (44)
Age at diagnosis	67 (8)	
BMI	23.7 (4.4)	
Stage 2 3 4		1 (0.8) 9 (7.1) 117 (92)
PS at inclusion 0 1 2		33 (26) 81 (64) 13 (10)
PD-L1 ^a^ >1% ≤1%		12 (57) 9 (43)
Number of metastatic sites 0 1 2 3 4 or more		9 (7.1) 32 (26) 42 (33.1) 25 (19.7) 19 (15)
Smoking No Actual Previous		15 (12) 18 (14) 94 (74)
COPD Yes No		23 (18) 104 (82)

^a^ missing data *n* = 106.

## Data Availability

The data presented in this study are available on request from the corresponding author.

## References

[1] Sung H., Ferlay J., Siegel R.L., Laversanne M., Soerjomataram I., Jemal A., Bray F. (2021). Global Cancer Statistics 2020: GLOBOCAN Estimates of Incidence and Mortality Worldwide for 36 Cancers in 185 Countries. CA Cancer J. Clin..

[2] Horn L., Spigel D.R., Vokes E.E., Holgado E., Ready N., Steins M., Poddubskaya E., Borghaei H., Felip E., Paz-Ares L. (2017). Nivolumab Versus Docetaxel in Previously Treated Patients With Advanced Non–Small-Cell Lung Cancer: Two-Year Outcomes From Two Randomized, Open-Label, Phase III Trials (CheckMate 017 and CheckMate 057). J. Clin. Oncol..

[3] Temel J.S., Greer J.A., Goldberg S., Vogel P.D., Sullivan M., Pirl W.F., Lynch T.J., Christiani D.C., Smith M.R. (2010). A Structured Exercise Program for Patients with Advanced Non-Small Cell Lung Cancer. J. Thorac. Oncol..

[4] Lutz S., Norrell R., Bertucio C., Kachnic L., Johnson C., Arthur D., Schwarz M., Palardy G. (2001). Symptom Frequency and Severity in Patients with Metastatic or Locally Recurrent Lung Cancer: A Prospective Study Using the Lung Cancer Symptom Scale in a Community Hospital. J. Palliat. Med..

[5] Planchard D., Popat S., Kerr K., Novello S., Smit E.F., Faivre-Finn C., Mok T.S., Reck M., Van Schil P.E., Hellmann M.D. (2019). Correction to: “Metastatic Non-Small Cell Lung Cancer: ESMO Clinical Practice Guidelines for Diagnosis, Treatment and Follow-Up”. Ann. Oncol..

[6] Borghaei H., Paz-Ares L., Horn L., Spigel D.R., Steins M., Ready N.E., Chow L.Q., Vokes E.E., Felip E., Holgado E. (2015). Nivolumab versus Docetaxel in Advanced Nonsquamous Non–Small-Cell Lung Cancer. N. Engl. J. Med..

[7] Brahmer J., Reckamp K.L., Baas P., Crinò L., Eberhardt W.E.E., Poddubskaya E., Antonia S., Pluzanski A., Vokes E.E., Holgado E. (2015). Nivolumab versus Docetaxel in Advanced Squamous-Cell Non–Small-Cell Lung Cancer. N. Engl. J. Med..

[8] Pamoukdjian F., Bouillet T., Lévy V., Soussan M., Zelek L., Paillaud E. (2018). Prevalence and Predictive Value of Pre-Therapeutic Sarcopenia in Cancer Patients: A Systematic Review. Clin. Nutr..

[9] Kiss N. (2016). Nutrition Support and Dietary Interventions for Patients with Lung Cancer: Current Insights. Lung Cancer.

[10] Raynard B., Pigneur F., Di Palma M., Deluche E., Goldwasser F. (2022). The Prevalence of CT-Defined Low Skeletal Muscle Mass in Patients with Metastatic Cancer: A Cross-Sectional Multicenter French Study (the SCAN Study). Support. Care Cancer.

[11] Martin L., Senesse P., Gioulbasanis I., Antoun S., Bozzetti F., Deans C., Strasser F., Thoresen L., Jagoe R.T., Chasen M. (2015). Diagnostic Criteria for the Classification of Cancer-Associated Weight Loss. J. Clin. Oncol..

[12] Lim S., Brown J.L., Washington T.A., Greene N.P. (2020). Development and Progression of Cancer Cachexia: Perspectives from Bench to Bedside. Sports Med. Health Sci..

[13] Ni J., Zhang L. (2020). Cancer Cachexia: Definition, Staging, and Emerging Treatments. Cancer Manag. Res..

[14] Bargetzi L., Brack C., Herrmann J., Bargetzi A., Hersberger L., Bargetzi M., Kaegi-Braun N., Tribolet P., Gomes F., Hoess C. (2021). Nutritional Support during the Hospital Stay Reduces Mortality in Patients with Different Types of Cancers: Secondary Analysis of a Prospective Randomized Trial. Ann. Oncol..

[15] Heudel P., Livartowski A., Arveux P., Willm E., Jamain C. (2016). ConSoRe: Un outil permettant de rentrer dans le monde du big data en santé. Bull. Cancer.

[16] Forrest L.M., McMillan D.C., McArdle C.S., Angerson W.J., Dunlop D.J. (2003). Evaluation of Cumulative Prognostic Scores Based on the Systemic Inflammatory Response in Patients with Inoperable Non-Small-Cell Lung Cancer. Br. J. Cancer.

[17] McMillan D.C. (2013). The Systemic Inflammation-Based Glasgow Prognostic Score: A Decade of Experience in Patients with Cancer. Cancer Treat. Rev..

[18] Gibney G.T., Weiner L.M., Atkins M.B. (2016). Predictive Biomarkers for Checkpoint Inhibitor-Based Immunotherapy. Lancet Oncol..

[19] Hopkins A.M., Rowland A., Kichenadasse G., Wiese M.D., Gurney H., McKinnon R.A., Karapetis C.S., Sorich M.J. (2017). Predicting Response and Toxicity to Immune Checkpoint Inhibitors Using Routinely Available Blood and Clinical Markers. Br. J. Cancer.

[20] Iivanainen S., Koivunen J.P. (2020). Possibilities of Improving the Clinical Value of Immune Checkpoint Inhibitor Therapies in Cancer Care by Optimizing Patient Selection. Int. J. Mol. Sci..

[21] Mountzios G., Linardou H., Kosmidis P. (2016). Immunotherapy in Non-Small Cell Lung Cancer: The Clinical Impact of Immune Response and Targeting. Ann. Transl. Med..

[22] Rounis K., Makrakis D., Tsigkas A.-P., Georgiou A., Galanakis N., Papadaki C., Monastirioti A., Vamvakas L., Kalbakis K., Vardakis N. (2021). Cancer Cachexia Syndrome and Clinical Outcome in Patients with Metastatic Non-Small Cell Lung Cancer Treated with PD-1/PD-L1 Inhibitors: Results from a Prospective, Observational Study. Transl. Lung Cancer Res..

[23] Ravasco P., Monteiro-Grillo I., Vidal P.M., Camilo M.E. (2004). Cancer: Disease and Nutrition Are Key Determinants of Patients’ Quality of Life. Support. Care Cancer.

[24] Sarhill N., Mahmoud F., Walsh D., Nelson K.A., Komurcu S., Davis M., LeGrand S., Abdullah O., Rybicki L. (2003). Evaluation of Nutritional Status in Advanced Metastatic Cancer. Support. Care Cancer.

[25] Khalid U., Spiro A., Baldwin C., Sharma B., McGough C., Norman A.R., Eisen T., O’Brien M.E.R., Cunningham D., Andreyev H.J.N. (2007). Symptoms and Weight Loss in Patients with Gastrointestinal and Lung Cancer at Presentation. Support. Care Cancer.

[26] Baracos V.E., Martin L., Korc M., Guttridge D.C., Fearon K.C.H. (2018). Cancer-Associated Cachexia. Nat. Rev. Dis. Primers.

[27] Wakefield L.M., Hill C.S. (2013). Beyond TGFβ: Roles of Other TGFβ Superfamily Members in Cancer. Nat. Rev. Cancer.

[28] Cohen S., Nathan J.A., Goldberg A.L. (2015). Muscle Wasting in Disease: Molecular Mechanisms and Promising Therapies. Nat. Rev. Drug Discov..

[29] Tisdale M.J. (2010). Are Tumoral Factors Responsible for Host Tissue Wasting in Cancer Cachexia?. Future Oncol..

[30] Argilés J.M., Busquets S., Stemmler B., López-Soriano F.J. (2014). Cancer Cachexia: Understanding the Molecular Basis. Nat. Rev. Cancer.

[31] Coss C.C., Clinton S.K., Phelps M.A. (2018). Cachectic Cancer Patients: Immune to Checkpoint Inhibitor Therapy?. Clin. Cancer Res..

[32] Fearon K., Strasser F., Anker S.D., Bosaeus I., Bruera E., Fainsinger R.L., Jatoi A., Loprinzi C., MacDonald N., Mantovani G. (2011). Definition and Classification of Cancer Cachexia: An International Consensus. Lancet Oncol..

[33] Flint T.R., Janowitz T., Connell C.M., Roberts E.W., Denton A.E., Coll A.P., Jodrell D.I., Fearon D.T. (2016). Tumor-Induced IL-6 Reprograms Host Metabolism to Suppress Anti-Tumor Immunity. Cell Metab..

[34] Basak E.A., Koolen S.L.W., Hurkmans D.P., Schreurs M.W.J., Bins S., Oomen de Hoop E., Wijkhuijs A.J.M., den Besten I., Sleijfer S., Debets R. (2019). Correlation between Nivolumab Exposure and Treatment Outcomes in Non–Small-Cell Lung Cancer. Eur. J. Cancer.

[35] Abe K., Shibata K., Naito T., Otsuka A., Karayama M., Maekawa M., Miyake H., Suda T., Kawakami J. (2021). Impacts of Cachexia Progression in Addition to Serum IgG and Blood Lymphocytes on Serum Nivolumab in Advanced Cancer Patients. Eur. J. Clin. Pharmacol..

[36] Roch B., Coffy A., Jean-Baptiste S., Palaysi E., Daures J.-P., Pujol J.-L., Bommart S. (2020). Cachexia-Sarcopenia as a Determinant of Disease Control Rate and Survival in Non-Small Lung Cancer Patients Receiving Immune-Checkpoint Inhibitors. Lung Cancer.

[37] Fujii H., Makiyama A., Iihara H., Okumura N., Yamamoto S., Imai T., Arakawa S., Kobayashi R., Tanaka Y., Yoshida K. (2020). Cancer Cachexia Reduces the Efficacy of Nivolumab Treatment in Patients With Advanced Gastric Cancer. Anticancer Res..

[38] Lee C.-S., Devoe C.E., Zhu X., Fishbein J.S., Seetharamu N. (2020). Pretreatment Nutritional Status and Response to Checkpoint Inhibitors in Lung Cancer. Lung Cancer Manag..

[39] Nishioka N., Uchino J., Hirai S., Katayama Y., Yoshimura A., Okura N., Tanimura K., Harita S., Imabayashi T., Chihara Y. (2019). Association of Sarcopenia with and Efficacy of Anti-PD-1/PD-L1 Therapy in Non-Small-Cell Lung Cancer. J. Clin. Med..

[40] Degens J.H.R.J., Dingemans A.C., Willemsen A.C.H., Gietema H.A., Hurkmans D.P., Aerts J.G., Hendriks L.E.L., Schols A.M.W.J. (2021). The Prognostic Value of Weight and Body Composition Changes in Patients with Non-small-cell Lung Cancer Treated with Nivolumab. J. Cachexia Sarcopenia Muscle.

[41] Pressoir M., Desné S., Berchery D., Rossignol G., Poiree B., Meslier M., Traversier S., Vittot M., Simon M., Gekiere J.P. (2010). Prevalence, Risk Factors and Clinical Implications of Malnutrition in French Comprehensive Cancer Centres. Br. J. Cancer.

[42] de van der Schueren M.A.E., Laviano A., Blanchard H., Jourdan M., Arends J., Baracos V.E. (2018). Systematic Review and Meta-Analysis of the Evidence for Oral Nutritional Intervention on Nutritional and Clinical Outcomes during Chemo(Radio)Therapy: Current Evidence and Guidance for Design of Future Trials. Ann. Oncol..

[43] Schiessel D.L., Baracos V.E. (2018). Barriers to Cancer Nutrition Therapy: Excess Catabolism of Muscle and Adipose Tissues Induced by Tumour Products and Chemotherapy. Proc. Nutr. Soc..

[44] Meuric J., Besnard I. (2012). Nutrition chez le patient adulte atteint de cancer: Quand doit-on proposer un conseil diététique personnalisé ?. Nutr. Clin. Métab..

[45] Senesse P., Bachmann P., Bensadoun R.J., Besnard I., Bourdel-Marchasson I., Bouteloup C., Crenn P., Goldwasser F., Guérin O., Latino-Martel P. (2012). Nutrition chez le patient adulte atteint de cancer: Textes courts. Nutr. Clin. Métab..

[46] Muscaritoli M., Arends J., Aapro M. (2019). From Guidelines to Clinical Practice: A Roadmap for Oncologists for Nutrition Therapy for Cancer Patients. Ther. Adv. Med. Oncol..

[47] van der Werf A., Langius J.A.E., Beeker A., ten Tije A.J., Vulink A.J., Haringhuizen A., Berkhof J., van der Vliet H.J., Verheul H.M.W., de van der Schueren M.A.E. (2020). The Effect of Nutritional Counseling on Muscle Mass and Treatment Outcome in Patients with Metastatic Colorectal Cancer Undergoing Chemotherapy: A Randomized Controlled Trial. Clin. Nutr..

[48] Cereda E., Cappello S., Colombo S., Klersy C., Imarisio I., Turri A., Caraccia M., Borioli V., Monaco T., Benazzo M. (2018). Nutritional Counseling with or without Systematic Use of Oral Nutritional Supplements in Head and Neck Cancer Patients Undergoing Radiotherapy. Radiother Oncol..

[49] Ravasco P., Monteiro-Grillo I., Vidal P.M., Camilo M.E. (2005). Dietary Counseling Improves Patient Outcomes: A Prospective, Randomized, Controlled Trial in Colorectal Cancer Patients Undergoing Radiotherapy. J. Clin. Oncol..

[50] Blackwood H.A., Hall C.C., Balstad T.R., Solheim T.S., Fallon M., Haraldsdottir E., Laird B.J. (2020). A Systematic Review Examining Nutrition Support Interventions in Patients with Incurable Cancer. Support. Care Cancer.

[51] Gomes F., Baumgartner A., Bounoure L., Bally M., Deutz N.E., Greenwald J.L., Stanga Z., Mueller B., Schuetz P. (2019). Association of Nutritional Support With Clinical Outcomes Among Medical Inpatients Who Are Malnourished or at Nutritional Risk: An Updated Systematic Review and Meta-Analysis. JAMA Netw. Open.

[52] Bachmann P., Bertrand A., Roux P. (2016). Prise en charge nutritionnelle dans les parcours de soins des cancers. Nutr. Clin. Métab..

[53] Cortellini A., Bersanelli M., Buti S., Cannita K., Santini D., Perrone F., Giusti R., Tiseo M., Michiara M., Marino P.D. (2019). A Multicenter Study of Body Mass Index in Cancer Patients Treated with Anti-PD-1/PD-L1 Immune Checkpoint Inhibitors: When Overweight Becomes Favorable. J. Immunother. Cancer.

[54] Dewys W.D., Begg C., Lavin P.T., Band P.R., Bennett J.M., Bertino J.R., Cohen M.H., Douglass H.O., Engstrom P.F., Ezdinli E.Z. (1980). Prognostic Effect of Weight Loss Prior Tochemotherapy in Cancer Patients. Am. J. Med..

[55] Hu Y., Shen J., Liu R., Feng Z., Zhang C., Ling L., Chen L. (2018). Prognostic Value of Pretreatment Prognostic Nutritional Index in Non-Small Cell Lung Cancer: A Systematic Review and Meta-Analysis. Int. J. Biol. Markers.

[56] Yan L., Nakamura T., Casadei-Gardini A., Bruixola G., Huang Y.-L., Hu Z.-D. (2021). Long-Term and Short-Term Prognostic Value of the Prognostic Nutritional Index in Cancer: A Narrative Review. Ann. Transl. Med..

